# A Short-Term Enteral Nutrition Protocol for Management of Adult Crohn’s Disease—A Pilot Trial

**DOI:** 10.3390/jcm13061663

**Published:** 2024-03-14

**Authors:** Levi Teigen, Matthew Hamilton, Austin Hoeg, Lulu Chen, Sharon Lopez, Amanda Kabage, Priyali Shah, Eugenia Shmidt, Byron Vaughn

**Affiliations:** 1 Department of Food Science and Nutrition, University of Minnesota, St. Paul, MN 55108, USA; priyalishah@gmail.com; 2 Microbiota Research and Therapeutics, University of Minnesota, Minneapolis, MN 55455, USA; hami0192@umn.edu; 3 Medical School, University of Minnesota, Minneapolis, MN 55455, USA; hoegx008@umn.edu; 4 Division of Gastroenterology, Department of Medicine, University of Minnesota, Minneapolis, MN 55455, USA; chen8124@umn.edu (L.C.); skunkel@umn.edu (S.L.); kabage@umn.edu (A.K.); eshmidt@umn.edu (E.S.); bvaughn@umn.edu (B.V.)

**Keywords:** Crohn’s disease, partial enteral nutrition, diet, microbiome

## Abstract

Crohn’s disease (CD) is often treated with either exclusive or supplemental enteral nutrition (EN) in pediatrics, but adult practice guidelines primarily focus on medications. Here, we demonstrate the feasibility of a 4-week semi-elemental-formula-based oral nutrition delivery program for managing adult CD (*n* = 4). Patients consumed ~66% of calories from the formula, a finding that might provide an improved calorie target for future trials. We identified *Flavinofractor* as the only differentially abundant genus, distinguishing post-intervention samples from pre-intervention samples. Findings from this pilot trial demonstrate the feasibility of a partial enteral nutrition protocol in adult CD management and contribute to the growing body of literature on the potential role of EN therapy in adults with CD.

## 1. Introduction

Over three million individuals in the United States have been diagnosed with inflammatory bowel disease (IBD) [1]. Crohn’s disease (CD), one of the two primary forms of IBD, is often treated with either exclusive or supplemental enteral nutrition (EN) in pediatrics [2]. In pediatrics, exclusive enteral nutrition is highly effective at inducing remission, with regular remission rates around 75% [3]. Clinically, children with CD often receive exclusive enteral diet therapy to induce remission. For adults, though the British Society of Gastroenterology consensus provides practice guidelines for exclusive EN to induce remission of CD in adults [4], both European and American practice guidelines primarily focus on medications [3,5]. This is, in large part, due to the limitations of palatability and acceptance among adults [6] surrounding enteral nutrition (EN) therapy’s role in managing IBD in adults.

Compliance represents a significant obstacle to exclusive enteral nutrition therapy for adult CD. Therefore, studies that have assessed EN therapy in adult CD populations often utilize partial enteral nutrition (PEN) protocols [7]. In children, a Crohn’s disease exclusion diet (CDED) coupled with PEN was better tolerated and led to a higher rate of sustained remission compared to exclusive EN [8]. Published protocols vary in the quantity of EN but have repeatedly shown success with at least 50% of calories from EN [7,9]. These studies also demonstrated success using either an elemental or polymeric formula. The definition of success, however, varied between the two studies assessing the ability of PEN to achieve clinical remission vs. measured relapse occurrence in individuals already in remission. These outcomes are essential to distinguish between as they have different clinical implications. The ability of PEN to achieve clinical remission is relevant therapeutically as a short-term intervention, whereas the ability of PEN to maintain remission frames PEN as more of a long-term maintenance therapy.

Regardless of its ultimate clinical role, however, the PEN mechanism of action still needs to be better understood. Changes in the gut microbiota, bowel rest, and improvements in nutritional status have all been proposed as potential mechanisms. Recent work by Pigneur et al. lends strong support to the role of EN in inducing remission, demonstrating increased mucosal healing compared to steroids, but also suggests changes to gut microbial composition as a possible mechanism of action [10]. Further, an overall improvement in nutritional status has also been hypothesized as a possible mechanism of action [11]. Here, we present results from a pilot trial to understand the therapeutic feasibility of a semi-elemental-formula-based oral nutrition delivery program targeting the provision of ≥80% of estimated calorie needs. Secondary outcomes included compliance and diet experience scores and changes in global health, physical and mental health scores, Crohn’s disease activity index (CDAI), and gut microbiome composition.

## 2. Methods

The institutional review board of the University of Minnesota approved this study (initial approval 5 April 2020). Informed consent was obtained from all participants. Participants were recruited from the Gastroenterology Clinic. The study design was an open-label, 4-week pilot-trial intervention utilizing self-administered diets. Participants communicated regularly with the study dietitian (LT) and other investigators to complete weekly questionnaires and support compliance. Patients > 18 years of age diagnosed with CD and moderate-to-severe disease were eligible for inclusion in this study. Inclusion criteria also included stable doses of CD therapy, defined as at least two weeks for corticosteroids, at least eight weeks for immunomodulators, at least four weeks for biologic therapy, and at least three weeks for oral aminosalicylates to isolate any potential effect of the intervention.

The enteral nutrition formula consumed was Peptamen^®^ 1.5 (vanilla; Nestle Health Science, Bridgewater, NJ, USA), a commercially available, semi-elemental formula. The formula was provided at or above the required volume to meet 80% of estimated calorie needs based on the Harris–Benedict equation. Crohn’s disease activity index (CDAI) and Harvey–Bradshaw index (HBI) scores were calculated at the baseline and end of the intervention period. Patient experience during the semi-elemental EN regimen was assessed weekly using an investigator-generated questionnaire. The PROMIS (v1.0) Emotional Distress–Depression—Short Form 4a and PROMIS (v1.2) Global Health Physical 2a were used to measure emotional distress and physical health, respectively. Patient–dietitian communication occurred weekly throughout the study period.

Dietary intake data consisted of three participant-recorded 24 h diet records completed each week throughout the study period. Participants were encouraged to record 2 weekdays and 1 weekend day each week. Participants received instruction from a trained and certified researcher in the University of Minnesota’s Nutrient Data System for Research (NDSR; University of Minnesota, Minneapolis, MN, USA) for accurate quantification and recording of dietary intake. The researcher reviewed completed records and obtained clarification from respective participants, as needed. NDSR nutritional analysis software (version 2022) was used for analysis of 3-day diet records. NDSR is a gold-standard for dietary recalls, providing high-quality data linked to the Nutrition Coordinating Center (NCC) food and nutrient database of over 18,000 foods and 178 nutrients [12,13].

Participants collected weekly stool samples (baseline (Day 0; before intervention); Day 7 ± 2 days; Day 14 ± 2 days; Day 21 ± 2 days; and Day 28 ± 2 days) in standard stool specimen collection kits. DNA was extracted from fecal samples using the DNeasy PowerSoil Kit (QIAGEN, Hilden, Germany), according to the manufacturer’s instructions. DNA samples were sequenced at the University of Minnesota Genomics Center targeting the V4 region of the 16S rRNA gene. The V4 hypervariable region of the 16s rRNA gene was amplified and sequenced at the University of Minnesota Genomics Center using the Illumina MiSeq v3 platform (Illumina, San Diego, CA, USA) and previously described primers and methods [14]. Sequence processing and analysis were performed using the mothur program version 1.41 [15] as previously described [16], adapted for the V4 hypervariable region.

### Statistical Analysis

Statistical analyses were performed using SAS version 9.4 (SAS Institute, Cary, NC, USA). Given the pilot nature of this trial, missing values were excluded from the analysis. Descriptive statistics were used to characterize the cohort. Paired *t*-tests were used to compare continuous outcome measures. The Shannon index of alpha diversity was calculated using mothur program 1.41. Ordination by principal coordinate analysis (PCoA) was completed using Bray–Curtis dissimilarity matrices. Linear discriminant analysis of effect size (LEfSe) was used to evaluate OTUs associated with pre- and post-intervention samples.

## 3. Results

### 3.1. Participants and Dietary Intake

Five participants were enrolled in this study with a median age of 36 years (range 20–49) and a length of CD of 16 years (range 4–30). All participants were Caucasian and consuming omnivorous diets at baseline. The median CDAI and HBI at baseline were 285 and 8, respectively. Most participants (4/5, 80%) tolerated and completed the intervention. However, one participant did not tolerate the PEN intervention and stopped consuming the formula after the first week. The subsequent analysis is limited to the four participants who completed the intervention.

The median age of the cohort of patients who completed the intervention (*n* = 4) was 37.5 (range 20–49) with a length of CD of 17.5 years (range 11–30). The cohort consisted of two males and two females with a median BMI of 21.8 kg/m^2^ (range 17.6–28.6) and median CDAI and HBI scores of 229 (range 91–314) and 8.5 (3–21), respectively. Medications remained stable over the intervention period, and PEN formula intake provided an overall median of 66% of calories (range 34–88%) and 0.9 g/kg/day of protein (range 0.7–1.3 g/kg/day). Average calorie intake over the intervention period was 32 kcal/kg/day; average protein intake was 1.4 g/kg/day; and average fiber intake was 6 g/day. No weight changes occurred during the intervention period.

### 3.2. Scores

Scores reflecting the overall experience on the PEN regimen improved from 3.25 to 4 (out of 5) from week one to week four but did not reach statistical significance (*p* = 0.2). Mean HBI and CDAI scores also trended towards an improvement (10 vs. 6.2 and 216 vs. 137, respectively) but similarly did not reach statistical significance. Mean PROMIS emotional distress scores trended towards an improvement from 8.25 to 6.5 (*p* = 0.06), while global health scores did not change.

### 3.3. Microbiota

**Figure 1:**
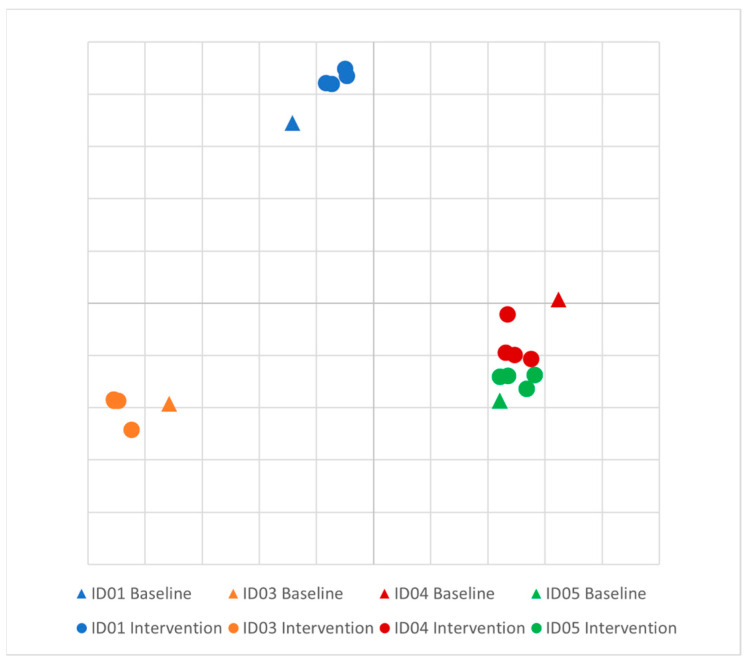
Principal coordinate analysis (PCoA) plot of longitudinal beta diversity by participant. Legend: Principal coordinate analysis of Bray–Curtis dissimilarities among participants. Five samples were collected from each healthy control over the course of the intervention, and each individual is differentiated by color.

**Figure 2:**
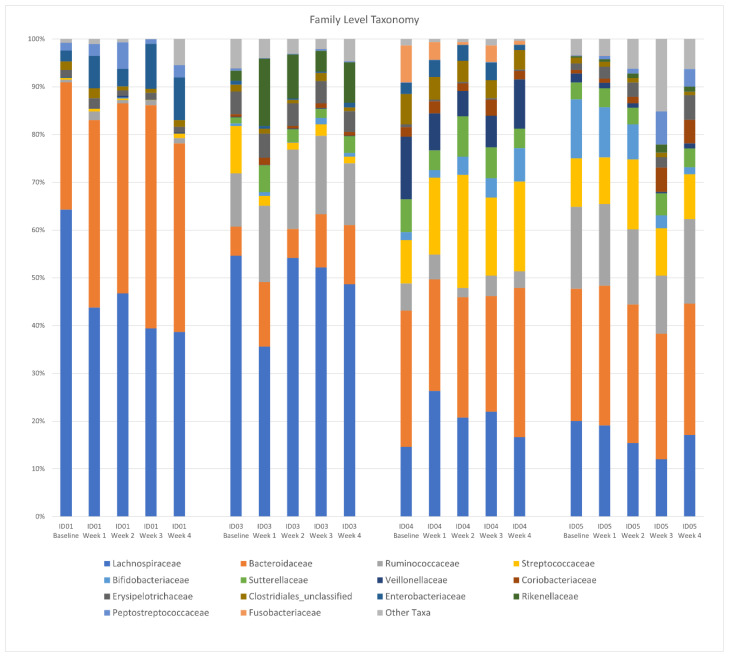
Longitudinal summary of gut microbiota composition by individual participant. Legend: Relative abundance of bacterial taxa in stool samples at the genus level. The *x*-axis represents weekly samples collected over the course of the intervention period by participant.

There was no difference in the Shannon diversity index between subjects (mean = 3.2) or between pre- and post-intervention (*p* = 0.1). Principal coordinate analysis (PCoA) of longitudinal beta diversity by participants (Figure 1) illustrates both the tendency for patient samples to cluster by themselves and the lack of a consistent change in beta diversity throughout the intervention. A longitudinal summary of gut microbiota composition by individual participants is presented in Figure 2. LEfSe analysis identified one differentially abundant genus, *Flavinofractor,* associated with post-intervention samples (LDA score 3.7).

## 4. Discussion

Here, we present the results of a pilot trial demonstrating the feasibility of a 4-week semi-elemental-formula-based oral nutrition delivery program for managing adult CD. Notably, although 80% of daily caloric intake was targeted, patients consumed ~66% of their daily calories from the formula, which might serve as a refined objective for future trials. By assessing the acceptability, adherence, safety, and efficacy of an EEN intervention, we aimed to determine whether this nutritional approach can serve as a viable alternative or adjunct therapy to conventional treatment modalities. While we observed trends toward clinical improvement around CD burden and emotional distress, this study was not powered to detect significant changes in these measures.

If the feasibility of this pilot study can be reproduced with higher-powered trials, a semi-elemental-formula-based oral nutrition program may offer several advantages over other therapies for Crohn’s disease management. Whether through the provision of easily digestible nutrients, potential reduction of antigenic load, promotion of mucosal healing, or modulation of gut microbiota, EN therapy presents a safe, viable, and efficacious option for CD management while reducing the treatment burden on patients living with this chronic disease [17]. Additionally, the potential benefits of improved nutritional status, reduced disease activity, and enhanced quality of life could significantly impact the overall well-being of individuals managing CD long term.

We identified *Flavinofractor* as the only differentially abundant genus distinguishing post-intervention samples from pre-intervention samples. Previous studies suggest changes to gut microbial composition as a possible mechanism of action of EN therapy [10]. While research on the *Flavinofractor* genus is limited, emerging evidence suggests its potential role in CD is related to dysbiosis, metabolic activity, and immune dysregulation.

Studies have shown alterations in the gut microbiota composition of individuals with CD through decreased bacterial diversity and changes in specific microbial taxa [18,19]. Specifically, some research has indicated that the *Flavinofractor* genus may be more prevalent in the gut microbiota of individuals with CD than in healthy individuals, leading to opportunistic dysbiosis [20]. Because *Flavinofractor* has also been associated with increased expression of pro-inflammatory cytokines, it has also been postulated that certain strains within this genus may contribute to immune dysregulation and further exacerbate the inflammatory process in CD by way of an overdriven immunomodulatory response [21]. With the *Flavinofractor* genus emerging as a potential agent in the complex ecosystem of the gut microbiota in individuals with CD, further research is required to understand better the exact role of this genus in CD pathogenesis, its impact on the immune response, and the potential therapeutic implications of its relative abundance, especially in the context of exclusive EN.

This short-term EN protocol contributes to the growing evidence supporting nutrition’s role in managing CD. Moreover, with promising trends in feasibility and patient outcomes, this pilot intervention could serve as a model for more extensive clinical trials. Additionally, continued investigations into the gut microbiota, including the *Flavinofractor* genus, hold promise for advancing our understanding of CD and potential targets for precision nutrition.

### Limitations

Our study has several limitations. This was a small pilot study and, therefore, may be inadequate to detect smaller but important differences in CD burden, tolerability, emotional distress, and global health scores. Another limitation is the lack of dietary control. While participants consumed most of their calories from the EN formula, non-EN nutrients were variable between participants, which may have confounded results in this small cohort. Future studies should seek to capture a period of the pre-intervention diet in order to support interpretation of changes in dietary intake associated with the intervention and potential implications for disease management. Additionally, individual CD presentation, along with gut microbial composition, are likely modified by diet. In sampling stool only once a week, we may have failed to minimize the inclusion of potential dietary outliers from a single meal, compared to having participants collect multiple stool samples each week. Finally, dietary data were collected using 24 h dietary records, which may have been subject to recall bias within individual participant responses.

## 5. Conclusions

Findings from this pilot trial suggest that a 4-week partial enteral nutrition protocol utilizing a semi-elemental formula is a feasible and acceptable option for patients with CD. Although 80% of daily caloric intake was targeted, patients consumed ~66% of their daily calories from the formula, which might serve as a refined objective for future trials. While we observed trends towards clinical improvement, this study was not powered to detect all relevant changes. Further research is needed with larger, more diverse cohorts, but these findings contribute to the growing body of literature on the potential role of EN therapy in adults with CD. We also identified *Flavinofractor* as the only differentially abundant genus distinguishing pre-intervention samples from post-intervention samples, which might suggest a potential role in CD. However, further work with a larger sample size, serial stool sample collection, and analysis time points is needed to elucidate the potential role of gut microbiota in the therapeutic efficacy of partial EN.

## Data Availability

The data presented in this study are openly available in the Bioproject database at http://www.ncbi.nlm.nih.gov/bioproject/1087401, reference number BioProject ID PRJNA1087401.
